# Capturing critical data elements in Juvenile Idiopathic Arthritis: initiatives to improve data capture

**DOI:** 10.1186/s12969-022-00745-z

**Published:** 2022-09-29

**Authors:** Meghan E. Ryan, Andrew Warmin, Bryce A. Binstadt, Colleen K. Correll, Emily Hause, Patricia Hobday, Alison Lerman, Shawn A. Mahmud, Mona M. Riskalla, Zachary Shaheen, Richard K. Vehe, Danielle R. Bullock, the Pediatric Rheumatology Care, Outcomes Improvement Network

**Affiliations:** 1 University of Minnesota and M Health Fairview Masonic Children’s Hospital, Minneapolis, USA; 2 Pediatric Rheumatology Clinic, Blank Children’s Hospital, 1206 Pleasant Street, Floor 2, IA, 50309 Des Moines, USA; 3 Cincinnati Children’s Hospital, Cincinnati, OH USA

**Keywords:** Juvenile idiopathic arthritis, Quality Improvement, Data Collection, Virtual visits

## Abstract

**Background:**

Documentation of critical data elements is a focus of the Pediatric Rheumatology Care and Outcomes Improvement Network to aid in clinical care and research for patients with juvenile idiopathic arthritis. We aimed to increase data capture for critical data elements and hypothesized that quality improvement methodology would improve data capture. We also hypothesized that data capture for all critical data elements would be lower for virtual visits compared to in-person visits.

**Methods:**

All visits for patients with JIA between 9/14/2020 and 12/31/2021 at the University of Minnesota were included. We assessed completeness of critical data element capture. Sixteen interventions with providers were conducted, including email reminders, individual discussions, group meetings, and feedback reports. We used statistical process control charts to evaluate change over time.

**Results:**

Baseline included 355 patient-visits: 221 (62%) in-person and 134 (38%) virtual with critical data elements entry ranging between 50 and 60%. Post-intervention included 1,596 patient-visits: 1,350 (85%) in-person and 246 (15%) virtual, with critical data elements entry reaching 91%. All providers improved data entry during this study. In-person visits had significantly higher data capture rates than virtual visits for all 4 critical data elements.

**Conclusion:**

We achieved our aim to increase critical data element documentation by focusing on provider buy-in, frequent reminders, and individualized feedback. We also found that collection of critical data elements occurred significantly less often with virtual visits than with in-person visits. Now that we improved capture of critical data elements, we can shift the focus to efforts aimed at improving outcomes for patients with juvenile arthritis.

## Background

Juvenile idiopathic arthritis (JIA) is the most common pediatric rheumatic condition with an estimated prevalence of 2 million children worldwide [1]. Rapid and sustained disease control is essential to avoid complications such as joint damage, limb growth discrepancies, and vision loss [2]. Based on supportive evidence for the use and efficacy of treat-to-target (T2T) in adult patients with rheumatoid arthritis, a T2T approach was recommended by an international task force of pediatric rheumatologists for all patients with JIA in order to achieve disease control [3, 4]. A T2T approach sets a target based on shared decision making between patients and providers that is measurable and reassessed over time. An open, single-arm, multicenter study in Germany used T2T for patients with polyarticular JIA and found a statistically significant improvement in disease remission as compared to those who did not use T2T [5].

The Pediatric Rheumatology Care and Outcomes Improvement Network (PR-COIN), an international quality improvement health network, adopted the T2T approach and, in partnership with an international task force of pediatric rheumatologists, developed an aim to increase the percentage of patients with JIA who have inactive to low active disease [4]. Disease activity was defined by the clinical juvenile disease activity score 10 (cJADAS10), a validated tool calculated in real-time using a patient/parent global assessment of wellbeing (PtGA), active joint count (AJC), and provider global assessment (PrGA) [6]. Disease severity cutoffs for oligoarticular and polyarticular JIA have been established, but not for other JIA subtypes [6, 7]. Steps to achieve PR-COIN’s aim of inactive to low active disease include (1) reliable data collection, (2) setting treatment targets with families and patients, and (3) utilizing clinical decision supports to inform treatment. In 2019, PR-COIN tracked the implementation of T2T across PR-COIN sites and reported that only 7 of the 16 sites (43%) were achieving high reliability for data collection, the base step in the process [8]. Therefore, efforts to improve data collection were a proposed focus for many sites.

In the spring of 2020, the COVID-19 pandemic created new challenges for the data collection process related to the rapid shift toward virtual visits and a pause in clinical research efforts. PR-COIN lead investigators raised concerns about the reliability and accuracy of the physical exam and assessment of disease activity in a virtual platform [9]. Goh, et al. proposed that additional research should identify ways to improve data collection in virtual visits [9].

The COVID-19 pandemic also provided an opportunity for PR-COIN to revitalize the T2T approach. A PR-COIN consensus conference held in the fall of 2020 recommended incorporation of patient goals when setting targets [10]. The original T2T goal was to improve disease activity. Other goals, such as improving patient arthritis pain, were also desired targets [10].

Initially, our group wanted to better understand arthritis pain as a target, so we began weekly data collection to determine the current state of arthritis pain relative to disease activity. However, manual chart review discovered a critical gap in data collection, with a baseline data collection rate of 52–61% for elements needed to calculate a cJADAS10 and the arthritis pain score. This raised questions about the reliability and accuracy of our data since quality data should be complete. Conclusions from incomplete data in-turn can lead to false beliefs and understandings of the results. We therefore adjusted our aim and focused on using methods to improve data capture for arthritis pain and other clinical variables for JIA disease activity. We hypothesized that quality improvement methodologies for critical data element (CDE) capture would improve data completeness. Critical data elements, as defined by PR-COIN, included two patient-reported outcomes (PROs), arthritis pain and PtGA, and two provider-assessed measures, PrGA and AJC. Our global aim was to improve CDE capture in the electronic medical record (EMR) such that the information could later be extracted and sent to the PR-COIN Registry. Our primary outcome measure was the proportion of CDE captured each week out of the total number of JIA patients seen. Secondarily, we hypothesized that in-person visit data entry would be better than virtual visit entry.

## Methods

### Patient selection

Patients with any JIA subtype, based on International League of Associations for Rheumatology (ILAR) criteria, seen for an in-person or virtual return visit were included. Telephone-only visits were excluded. Other exclusions included new patient visits, patients without a clear diagnosis of JIA, and patients with arthritis related to other diseases such as systemic lupus erythematosus, mixed connective tissue disease, or scleroderma. Three patients diagnosed with systemic JIA without significant arthritis and who had smoldering macrophage activation syndrome were also excluded.

### Stakeholders

The stakeholders included pediatric rheumatologists and pediatric rheumatology fellows, which we denote as providers in this paper. We had between 6 and 10 providers collecting data during the 68 weeks. Variation in the number of providers over the course of the study related to personal leave, retirement, and new hires.

### Outcome measures

The primary outcome measure was the proportion of CDE captured in an extractable form within the EMR out of the total number of JIA patients seen each week. Our institution uses Epic, an EMR that contains a data entry tool called a SmartForm. The SmartForm allows data to pull into clinic notes and to be extracted for use into registries such as the PR-COIN Registry. The SmartForm contains standardized data components chosen by PR-COIN which includes arthritis pain (0–10 scale with 1.0 increments), PtGA (0–10 scale with 0.5 increments), AJC, and PrGA (0–10 scale with 0.5 increments). Secondary outcome measures included the difference between in-person and virtual visit data entry.

### Data source and collection

We screened problem lists for all patients scheduled with pediatric rheumatology between 9/14/2020-12/31/2021 at our institution. For patients who met inclusion criteria, we completed a manual chart review of both visit documentation and CDE found in the SmartForm. Data found in the provider note but not in the SmartForm did not count in the numerator as these data could not be extracted in an automated way. However, these data elements documented in provider notes but not in the SmartForm were used for feedback during individual provider meetings and provider reports which were two of our interventions detailed below.

For in-person visits, arthritis pain and PtGA were filled out by patients and/or parents/guardian on paper intake forms. These two CDE needed to be verbally asked by the providers during virtual visits. Active joint count and PrGA were assessed by the providers for both visit types. All data entered in the SmartForm were dependent on provider entry.

### Interventions

**Fig. 1:**
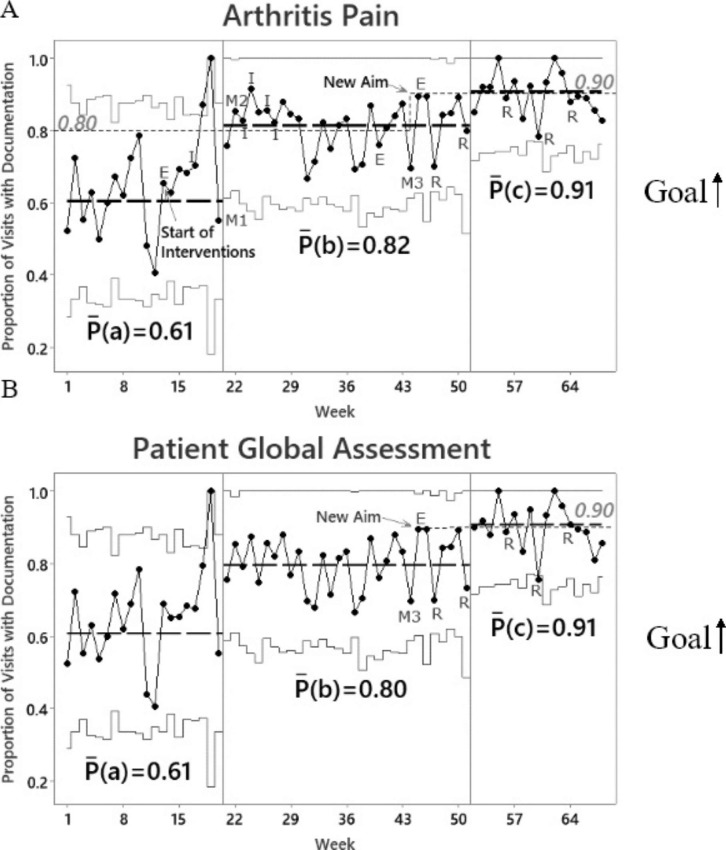
**Control P-Charts of Documentation for Patient-Reported Outcome Measures.** Data points are charted over time. Dashed lines represent means calculated based on 12 consecutive points, if available, for each interval, P(a) = weeks 1-12, P(b) = weeks 21-32 and P(c) weeks 52-63. Dotted lines represent aims. Solid lines represent control limits. Upward shifts occurred at weeks 21 and 52 for both **A** and **B**. E = email; I = individual discussions; M= group meetings; R = feedback reports

**Fig. 2:**
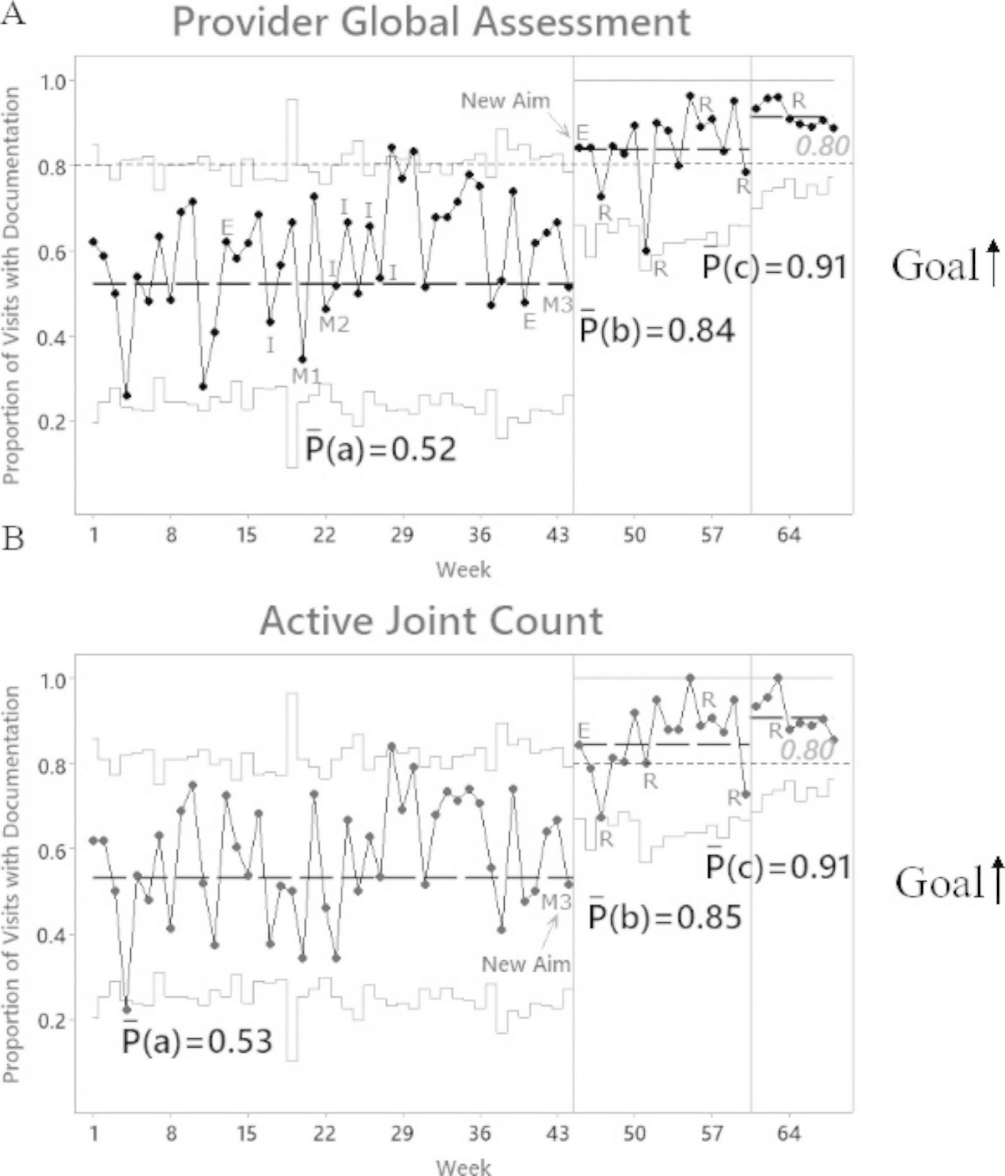
**Control P-Charts of Documentation for Provider-Assessed Measures.** Data points are charted over time. Dashed lines represent means calculated based on 12 consecutive points, if available, for each interval, P(a) = weeks 1-12, P(b) = weeks 45-56 and P(c) weeks 61-68. Dotted lines represent aims. Solid lines represent control limits. Upward shifts occurred at weeks 45 and 61 for both **A** and **B**. E = email; I = individual discussions; M= group meetings; R = feedback reports

Sixteen interventions were tested beginning at week 13 as illustrated in Figs. 1 and 2. The initial intervention was an email asking providers to document arthritis pain and PrGA in ≥ 80% of visits. Baseline data collection prompted meeting with an individual provider to assess barriers to data collection. A few weeks later, we held a group meeting (M1) to discuss weekly data collection rates via a run chart and to have a formalized discussion about our aim for ≥ 80% collection for arthritis pain and PrGA. At a second meeting (M2), we presented the evidence-base for T2T to provide relevance for CDE collection and learned that providers would like frequent and individualized feedback. Over the next several weeks, individual meetings with providers occurred to review each provider’s weekly data collection rate and to discuss provider-specific processes, barriers, and suggestions for data collection.

In a third virtual group meeting (M3), we discussed themes from individual provider meetings, reviewed our improvements in data collection, revised our aims, and elicited group input on preferences for feedback reports. We established new aims to document arthritis pain and PtGA for ≥ 90% of visits and PrG and AJC for ≥ 80% of visits. New feedback reports included group and provider-specific data on the percent of patient-visits each week with arthritis pain ≤ 3 and a breakdown of disease activity based on the cJADAS10 cutoffs for oligoarticular and polyarticular JIA. Other JIA ILAR subtype’s disease activity was reported by PrGA, with inactive being equal to zero and active ≥ 0.5.

### Analysis

Minitab 20.3 software was used. Statistical process control charts evaluated change over time. Baseline included the first 12 data points which were used to calculate the first centerline, the thick dashed line P(a), as shown in Figs. 1 and 2. Per control chart rules, a shift in the centerline occurs when there is a statistically significant change in the process which is defined by ≥ 8 consecutive points above or below the centerline. New centerlines were then calculated based on 12 data points, if available.

Control charts were initially separated into virtual and in-person visits for each CDE, in order to analyze how interventions impacted documentation based on visit type. However, the proportion of virtual visits decreased substantially in the post-intervention period, which made virtual visit control chart limits widely variable. Therefore, final control chart analysis incorporated the combination of virtual and in-person visits.

Inferential statistics included confidence intervals, ANOVA testing, and two-sample t-test. We separated the visit type comparisons into 3 different phases to minimize data bias. ANOVA testing was conducted on the weekly ratios of virtual to total visits for baseline (phase 1), post-intervention weeks 13–32 (phase 2) and post-intervention weeks 33–68 (phase 3). Two-sample t-tests were used to compare the weekly proportions of in-person and virtual visit data capture by phase.

## Results

### Visit characterization

We reviewed a total of 1,953 patient-visits. Table 1 provides baseline and post-intervention totals and weekly statistics.

**Table 1:** Characteristics of JIA visit types

	Total visits	Weekly Visits		
		Mean	Standard deviation	Range
Baseline: Week 1–12				
Total	355	29.6	6.6	21–46
In-person	221 (62.2%)	18.4	8.8	7–41
Virtual	134 (37.8%)	11.2	5.9	4–25
Post-intervention: Week 13–68				
Total	1598	28.5	7.6	12–46
In-person	1352 (84.6%)	24.1	7.5	9–44
Virtual	246 (15.4%)	4.4	5.1	0–20

### CDE capture

Baseline data entry of CDEs for all visit types ranged between 52 and 61%. About half of the providers had inconsistent or no data collection at baseline, and no single provider collected 90–100% of CDEs each week at baseline. Three providers were using an old SmartForm for data entry prior to their individual meetings, but all ultimately adopted the new SmartForm. All providers improved data collection during this project, and by the end most achieved 90–100% data collection consistently. The most frequently reported challenges with CDE collection were the time required and individual provider concerns about the organization and navigability of the SmartForm.

Email reminders and group meetings (M1 and M2) led to an improvement in arthritis pain and PtGA data entry to ≥ 80%, as shown by the upward shift at week 21 (Fig. 1 A-B). AJC and PrGA documentation shifted upward later at week 45 (Fig. 2 A-B) which occurred after intervention M3. Additional shifts occurred after providers received monthly feedback reports with patient pain and disease activity scores (Figs. 1 and 2).

### In-person versus virtual care

Significant differences (p-value < 0.001) in the proportion of virtual visits in phase 1 (mean 0.39, 95% CI 0.27–0.51) and phase 2 (mean 0.31, 95% CI 0.24–0.38) were found when compared to phase 3 (mean 0.06, 95% CI 0.04–0.07). Two-sample t-tests showed that regardless of phase, in-person visits had significantly higher data capture rates than virtual visits for all four CDEs (Table 2).

**Table 2:** Two-Sample T-Tests for CDE Collection Ratios Between In-person and Virtual Visits

	Phase 1 (week 1–12)		Phase 2 (week 13–32)		Phase 3 (week 33–68)	
	Mean (Standard deviation)	p-value	Mean (Standard deviation)	p-value	Mean (Standard deviation)	p-value
Arthritis Pain						
In-person Virtual	0.73 (0.13) 0.38 (0.19)	< 0.001*	0.86 (0.11) 0.60 (0.27)	0.001*	0.88 (0.07) 0.39 (0.42)	< 0.001*
Patient Global Assessment						
In-person Virtual	0.73 (0.12) 0.39 (0.18)	< 0.001*	0.85 (0.11) 0.56 (0.28)	< 0.001*	0.87 (0.08) 0.36 (0.41)	< 0.001*
Active Joint Count						
In-person Virtual	0.69 (0.16) 0.26 (0.16)	< 0.001*	0.70 (0.12) 0.27 (0.24)	< 0.001*	0.81 (0.16) 0.34 (0.39)	< 0.001*
Provider Global Assessment						
In-person Virtual	0.65 (0.15) 0.26 (0.19)	< 0.001*	0.72 (0.14) 0.32 (0.23)	< 0.001*	0.81 (0.14) 0.39 (0.40)	< 0.001*

## Discussion

Reliable data collection is necessary to accurately assess outcomes but has been a major challenge at many PR-COIN sites, including ours. Aided by quality improvement processes, we achieved our aim of increasing CDE documentation for arthritis pain, PtGA, PrGA, and AJC by first characterizing our data collection and then focusing on provider buy-in, frequent reminders, and individualized feedback.

We targeted provider buy-in during group and individual provider meetings by reviewing the relevance of data completeness for research and clinical practice and by discussing barriers and solutions for data capture. Through assessing individual provider barriers, we were able to assist in strategies that changed their workflow and increased data entry completeness. With new workflows established, data entry became a habit and resulted in continued success which we expect will be sustained in the long term. Provider interest in personalized feedback regarding patient outcomes, specifically patient disease activity scores, as opposed to the provider’s ability to capture data seemed to motivate documentation as well.

We performed interventions concurrently so that we could quickly achieve our aim. Overlapping interventions are common in quality improvement work, but they do limit one’s ability to determine effects of each individual intervention. It takes several data points after one intervention to determine the effect of that intervention. In determining which interventions our group would pursue, we explored different levels of intervention reliability -- the probability that a process will perform a desired task – and hence the likely impact of an intervention. Low reliability interventions have a lower likelihood for sustainability and include interventions in which people are simply asked to remember to do a task. Most of our intervention reliabilities were low, which is a limitation of our study. High reliability interventions are more likely to achieve and maintain a desired task as would be achieved with computer-automated interventions, such as patient entered PROs filled out on tablets and pulled into the SmartForm automatically. We discussed and desired high reliability interventions but could not execute them due to limited technology support and resources, especially during the pandemic. Low reliability interventions used frequently and concurrently increases the probability of success, which was the strategy we ultimately applied. When comparing our interventions, we speculated that email reminders resulted in a smaller effect and were less sustainable as a primary intervention compared to individual meetings or feedback reports. Ongoing challenges with CDE collection include the time required to collect and enter data and individual provider concerns about the organization and navigability of the SmartForm, items that may benefit from higher reliability interventions.

Our work found that improved documentation of PROs occurred several weeks prior to improvement in provider-assessed measures, despite initially targeting arthritis pain and PrGA. We postulate that multiple factors contributed to these differences, including the use of an intake form for PROs, appearance and position of CDEs on the SmartForm, time commitment, and provider level of comfort associated with each CDE. Similar to the majority (84%) of PR-COIN sites, we use paper intake forms to collect PROs during in-person visits [9]. Parallel improvements were observed for arthritis pain and PtGA data capture, which we hypothesize was due to the use of an intake form and the location of these PROs at the top of the SmartForm. The difference in time required to obtain two PROs rather than just one is minimal, which likely explains why PtGA improved in parallel with arthritis pain despite our not targeting PtGA initially.

Improvement in collection of PrGA and AJC occurred at week 45, despite targeting PrGA at week 13 and AJC at week 43. Providers disclosed having no formal education on how to rate a PrGA and discomfort with assessing PrGA and AJC virtually. The lack of concordance of interrater scoring for PrGA has been demonstrated previously and highlights the need for systematic training and well-defined guides for rating PrGA [11]. We postulate this intervention would result not only in more reliable data collection but also a more accurate assessment of the patient’s clinical status. Additionally, validation and standardization of PrGA and AJC virtually is needed.

Generalizability is a limitation since institutions have different processes, resources, EMRs, and virtual visit frequencies. For example, our utilization of the SmartForm was the major tool for tracking the CDEs. Not all sites have the same EMR system or SmartForm. Additionally, resource differences, such as paper intake versus electronic intake forms, would likely contribute to differences in data collection. Even though these processes may differ, the concepts of tracking data and providing individual feedback can be generalized.

Another limitation included the decline in virtual visit numbers over time which may have skewed the data when comparing virtual to in-person data collection. We attempted to compensate for this by splitting data into phases.

### Future directions

Reliance on manual chart review was key to this project’s success. In the longer term, a more automated process is needed. Ideally, we would have electronic questionnaires that input PROs directly into the SmartForm. Such a platform for data entry would likely improve data capture for both in-person and virtual visit PRO collection. The SmartForm is intended to facilitate extractable data, which is pushed into the PR-COIN Registry, a centralized database whose output is similar to the feedback reports we have been manually generating. Feedback reports created by PR-COIN and presented to individual sites could facilitate improved data entry for all metrics, including provider-assessed measures. Systematic training for PrGA and validating virtual visit assessment of critical data elements is also a necessary next step to improve both documentation and validity data.

## Conclusion

In conclusion, our work highlights the necessary building blocks for improved outcomes in patients with JIA. Reliable CDE collection is an essential step to utilizing validated disease activity scores to inform T2T strategies and ultimately improving patient outcomes.

Tables.

## Data Availability

The datasets used and/or analyzed during the current study are available from the corresponding author on reasonable request.

## Abbreviations

AJC: Active Joint Count.
CDE: Critical Data Elements.
JIA: Juvenile Idiopathic Arthritis.
PR-COIN: Pediatric Rheumatology Care and Outcomes Improvement Network.
PrGA: Provider Global Assessment.
PROs: Patient Reported Outcomes.
PtGA: Patient Global Assessment of Overall Wellbeing.
T2T: Treat-to-target.
